# On the Modeling and Verification of Collective and Cooperative Systems

**DOI:** 10.3389/frobt.2022.866649

**Published:** 2022-06-27

**Authors:** Alessandro Aldini

**Affiliations:** Department of Pure and Applied Sciences, University of Urbino Carlo Bo, Urbino, Italy

**Keywords:** collective adaptive systems, social networks, process algebra, model checking, temporal logics

## Abstract

The formal description and verification of networks of cooperative and interacting agents is made difficult by the interplay of several different behavioral patterns, models of communication, scalability issues. In this paper, we will explore the functionalities and the expressiveness of a general-purpose process algebraic framework for the specification and model checking based analysis of collective and cooperative systems. The proposed syntactic and semantic schemes are general enough to be adapted with small modifications to heterogeneous application domains, like, e.g., crowdsourcing systems, trustworthy networks, and distributed ledger technologies.

## 1 Introduction

Cooperation activities and collective behaviors are widespread phenomena in several environments, ranging from nature to human social relationships and artificial systems. Therefore, they have cross-cutting implications in different specific fields of knowledge, including, just to cite a few, biology (Crall et al., 2019; Glen et al., 2019; Romanov et al., 2022), sociology (Takano and Ichinose, 2018; Will et al., 2020), and robotics (Dai et al., 2016; Rausch et al., 2020; Mehmood et al., 2021). Although different levels of abstraction are involved, information sharing mechanisms form the base for the evolution of biological, social, and engineering systems exhibiting the behaviors specified above. In particular, the efficiency of these mechanisms determines not only the success of individuals but also the fitness of systems of communities of such individuals. This is even more critical whenever:

1. The systems need to be adaptive with respect to dynamically changing environments;
2. A multiplicity of different types of agents collaborate (or compete) to engage in community decision processes (or to achieve individual goals to survive and emerge);
3. Complex tasks are interleaved with frequent mutual interactions.

In this respect, one of the main aspects to pay attention to is given by the communication and cooperation models, with a specific emphasis on the information exchange policies, the allocation of tasks and of resources, the synchronization of activities converging to group goals. Moreover, it is worth distinguishing the nature and use of the information that may be subject to exchange, which can derive from the external environment, be processed by every agent in isolation, and/or represent community-based shares.

All these considerations play a role when devising techniques to model, verify, and develop collective and cooperative systems - see, e.g., De Nicola et al. (2020) and the references therein for a comprehensive overview. In this paper, we concentrate on the issues related to the formal modeling and verification of such systems. To this aim, we propose a general-purpose process algebraic framework that can be instantiated to the various and heterogeneous application domains surveyed above. The basic ingredients of this framework focus on the specification of the autonomous behavior of the agents, the handling of data collected from the environment and shared with the neighbours, the mode of interaction within communities of agents, the topology of the interacting communities, the dynamically changing external environment and system configuration. The flexibility of the approach is the main contribution provided by the framework, which makes it adequate to model and verify both natural and socially collective systems (including social networks as well as crowdsourcing systems), and artificial networks (including P2P and GRID systems, multi-agent systems, and sensor networks).

The kernel of the specification language is based on process algebra and relies only on a few, basic set of operators for the description of the behavioral pattern of agents in isolation. The syntax is left as simple as possible and abstracts away from the overwhelming details of standard parallel composition operators, thus making the process of composing even large networks of agents easy and scalable.

The semantics of the language encodes the mode of communication among agents, through rule schemes that support flexibility and adaptiveness with respect to the specific application domain of interest. Moreover, the framework includes the capability of grouping agents into dynamic communities, and to model local information stored by agents as well as global information shared within a given community of agents. Such an agent/community-oriented modeling framework is equipped with a temporal logic for the specification of properties of agents, communities, and networks. Thus, the flexibility of the modeling paradigm is inherited also by the property specification framework, enabling the definition of various property patterns, ranging from safety to performance.

The rest of the paper is organized as follows. In the next section, the basic syntax and semantics of the modeling framework are presented, by emphasizing the way in which customized semantics rules can be devised depending on the application domain. Section 3 defines the temporal logic for property specification. The applicability of this framework to various application domains is illustrated in Section 4 via some real-world references and examples. Finally, a discussion on related and future work is the topic of Section 5.

## 2 Modeling Agents and Networks

A key aspect for simplifying as much as possible the description of complex networks of agents is the clear separation between the description of each agent in isolation and the definition of the network of agents. This is even more crucial for formal paradigms like process algebra, which are typically based on a set of algebraic operators that join together the two levels of descriptions surveyed above, i.e., the agent level and the network level.

The separation of concerns between the definition of the system topology and of the behavioral pattern of the agents forming such a topology is a typical approach of architectural description languages–see, e.g., Aldini et al. (2010)—and is indeed motivated by usability and scalability issues. Therefore, we base the modeling framework on such a separation.

### 2.1 Modeling Behavioral Patterns and Agents

As a first step, we start with the presentation of a basic calculus–see, e.g., Fokkink, (2007)—for the description of the isolated behavior of sequential processes.

Let *Act* be the set of actions, ranged over by *a*, *b*, … , including also the special internal action *τ*. The set L of process terms of the basic calculus for sequential processes is generated through the following syntax:

$$\begin{array}{c} P\;⩴\;\underline{0}|a.\;P|P+P|B \end{array}$$

where we have the constant $\underline{0}$ for the inactive process, the classical algebraic operators for prefix and nondeterministic choice, and a constant based mechanism for expressing recursive processes, such that a set of constants defining equations of the form $B\overset{def}{=}P$ is assumed. As standard, we consider only guarded and closed process terms. The semantics of process terms is expressed in terms of labeled transition systems.

Definition 1. *A labeled transition system (LTS) is a tuple* (*Q*, *q*_0_, *L*, *R*)*, where* *Q* *is a finite set of states* (*with* *q*_0_ *the initial one*)*,* *L* *is a finite set of labels, and* *R* ⊆ *Q* × *L* × *Q* *is a finitely-branching transition relation.*

As a shorthand, (*q*, *a*, *q*′) ∈ *R* is denoted by $q\overset{a}{\to }q^{\prime }$ . Then, the behavior of process term *P* is defined by the smallest LTS (L,P,Act,R) , where the transitions in *R* are obtained through the application of the operational semantics rules of Table 1. The *prefix* rule is at the base of the sequential behavior of processes, stating that *a*. *P* executes *a* and then behaves as *P*. The two *choice* rules express the nondeterministic choice between *P*_1_ and *P*_2_. The winning process proceeds with its execution, thus disabling once and for all the other one. The *recursion* rule establishes that the process term named *B* and defined as *P*, behaves as *P* itself; naming enables the definition of recursive behaviors.

**TABLE 1:** Semantics rules of the basic calculus.

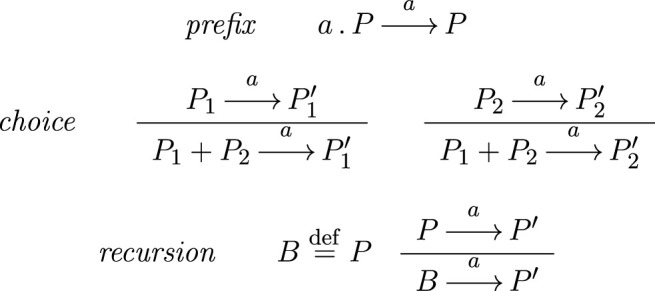

Example 1.

As a first running example, we consider a social network in which various agents contribute to the spreading of (possibly fake) news. A detailed version of this system is modeled and analyzed in Aldini, (2022), by using a formal framework that turns out to be an instance of that proposed in this work. Here, we start considering a simple, process term:

$$F\overset{def}{=}nbr.\left(re-evaluate.F+forget.\underline{0}\right)$$

*which models the behavior of a fact checker in such a network. Action* *nbr* *denotes the gathering of shared news from the neighbourhood, action* *forget* *expresses that any shared news is forgotten once and for all, and action* *re*-*evaluate* *denotes that the process of news evaluation is repeated again.*

As another running example, we will consider a trustworthy network of communities, where agents exchange services and the interactions among agents are enabled/disabled by trust/distrust relations. A detailed version of this system is modeled and analyzed in Aldini, (2018) through an alternative framework that, similarly as above, is generalized by the current proposal. Here, we start considering a simple, process term:

$$T\overset{def}{=}snd\text{\_}\mathrm{req}.\left(rec\text{\_}\mathrm{acc}.T+rec\text{\_}\mathrm{ref}.T\right)+leave\text{\_}\mathrm{com}.\underline{0}$$

*describing the behavior of a trustor, which may ask for services from the unique provider operating in the community to which the trustor belongs. Action* *snd*_*req* *denotes a service request sent to such a provider, which in fact represents the trustee subject of trust evaluation by the trustor; the request can be either accepted* (*action* *rec*_*acc*) *or refused* (*action* *rec*_*ref*)*. Alternatively, the trustor may decide to abandon the community* (*action* *leave*_*com*)*.*

In the following, an *agent* is any instance of a given process term *P*, which is referred to as the behavioral type (or pattern) of the agent. In other words, an agent represents an element exhibiting the behavior associated with a process term. Agents are associated with a unique identity, which in the following we denote with a natural number for the sake of simplicity. Moreover, each agent is equipped with a local data repository, used to store local parameters as well as data retrieved from sensors or received from the neighborhood. Such a repository is represented as a set of local atomic predicates. By assuming a standard first-order logic interpretation, predicates are of the form *v* = *d*, with *v* ∈ *VNames* a local variable and *d* a value of the corresponding domain.

Formally, an agent is described by a triple of elements ⟨*id*, *P*, *V*⟩, where:

• id∈N is the identity of the agent;
• process term P∈L is its behavioral type;
• function *V*: *VNames*↦*D* is the mapping from local variables to values in their corresponding domain *D*.^1^

Given the triple ⟨*id*, *P*, *V*⟩, as a shorthand we sometimes use the classical dot notation *id*. *P* to denote the local behavior of agent *id*, *id*. *a* to denote an action *a* enabled by the local behavior *P* of agent *id*, and *id*. *v* to denote the value *V*(*v*) of the local variable *v* in the local data repository of agent *id*.

Example 2. *A fact checker named* *id* *of behavioral type* *F* *is described by the triple* ⟨*id*, *F*, *V*⟩*. The local variables are:* *type* *, which expresses the level of susceptibility of the agent to accept shared news;* *accept* *, which is a Boolean modeling whether the news is accepted and in turn shared by the agent;* *threshold* *, which expresses the minimum number of neighbours that must share the same news in order to consider the news for acceptance.*

*A trustor named* *id* *of behavioral type* *T* *is described by the triple* ⟨*id*, *T*, *V*⟩*. The local variables are:* *α* *and* *β* *, reporting the number of accepted (respectively, refused) requests, and* *θ* *, which represents the trust threshold employed by the trustor for the trust-based evaluation of the trustee.*

While it is easy to see that the agent local semantics is given by the semantics of its behavioral type, it is less obvious to determine the agent’s behavior in the context of the environment. Such a context affects also the updates applied by the agent to its local repository. Therefore, we need to define formally the interaction semantics for a network of communicating agents.

### 2.2 Modeling Networks of Interacting Agents

A network is a set of agents, which are grouped to form (possibly dynamic) communities. Basically, direct interactions among agents are possible only within the same community. However, each agent, in general, may belong to several different communities at the same time. Similarly as in the case of single agents, each community is associated with a global data repository, storing data that can be shared by all the community participants. Such a repository is modeled as a set of global atomic predicates of the form *w* = *d*, with *w* ∈ *WNames* ^2^ a global variable and *d* a value of the corresponding domain.

Formally, a network is a triple of elements ⟨S,G,W⟩ , where:

• S is the finite set ${⋃}_{i=1}^{n}\left\langle {id}_{i},P_{i},V_{i}\right\rangle$ of *n* agents in the network, such that *id*_*j*_ ≠ *id*_*k*_ for every pair of indexes *j*, *k*;
• function $G:CNames\to 2^{N}$ maps every community (with *CNames* being the set of community names) to the set of agents identities forming it, thus representing the network topology;
• function W:CNames→(WNames↦D) maps every community to the related mapping from global variables to values in their corresponding domain.

The semantics of a network and, in particular, the way in which the constituting agents cooperate and evolve, depend on requirements of the specific scenario under consideration. Hence, (almost all) the rules we are going to introduce are actually schemes of rules including customizable elements. The first, fundamental modeling choice is related to the mode of execution, for which we distinguish two classical, alternative cases: *asynchronous* mode, where every agent may execute an autonomous action while all the others remain idle, and *synchronous* mode, where all the agents involved simultaneously execute one of their enabled actions.

The general semantic rule scheme for the asynchronous mode is as follows:

$$\left(async\right)\;\frac{P\overset{a}{\to }P^{\prime }\;\wedge \;cond}{\langle \left\{\langle id,P,V\rangle \right\}\cup S,G,W\rangle \overset{a}{\to }\langle \left\{\langle id,P^{\prime },V^{\prime }\rangle \right\}\cup S,G,W^{\prime }\rangle }$$

where the side condition cond stands for a Boolean formula composed of logical predicates over any combination of identities, communities, local variables, and global variables taken from the current network triple ⟨{⟨id,P,V⟩}∪S,G,W⟩ , and stating whether the action *a* offered by the local behavior *P* of agent *id* is enabled in the network environment. Hence, the side condition is actually of the form $cond\left(a,id,V,S,G,W,V^{\prime },W^{\prime }\right)$ . The additional terms *V*′ and $W^{\prime }$ depend on *V* and W , respectively, and represent their updated versions by virtue of the execution of the action *a*. More details about these terms and the definition of the side condition will be provided through examples. Notice that, for the sake of readability, in the rule scheme above and in the following ones, the side condition cond is reported without making the list of arguments explicit.

Example 3. *We present three typical formats for the atomic predicates that can be combined through logical connectives to define the side condition* cond *in the rule scheme* *async* *:*

1. *id*. *v*⋈*k* *, with* ⋈ *any arithmetic comparison operator and* *k* *a scalar value belonging to the domain of the local variable* *v* *: such a condition is purely local as it does not depend on the context in which agent* ⟨*id*, *P*, *V*⟩ *operates;*
2. ∃G∈CNames:id∈G(G)∧id.v⋈(W(G))(w) *, which compares the local variable* *v* *of the agent to the global variable* *w* *of a community* *G* *to which the agent belongs* (id∈G(G)) *;*
3. *id*. *v*⋈*f*(*X*)*, where* *f* *is a scalar function (e.g., min, sum, count) applied to a set* *X* *of local/global variables filtered in a certain way, and returning a value belonging to the domain of variable* *v* *.*

*Analogous patterns can be envisioned by defining conditions over* *id* *rather than over* *id*. *v* *.*

Later on we will show some exemplifying conditions specifically adapted to the application domains of interest. As stated above, the rule scheme *async* expresses also potential side effects of the execution of the action *a* over the local variables of the agent *id* and/or over the global variables of the network. Formally, the terms *V*′ and $W^{\prime }$ represent the updated versions of the terms *V* and W , respectively. On one hand, they may be equal to *V* and W , respectively, to express that no change occurs. On the other hand, they may be defined in terms of updates occurring in *V* and W . To this aim, in the following examples we will use the standard notation *s*′ = *s*[*x*↦*d*] to express a mapping *s*′ equal to *s* in every point but *x*, where *s*′(*x*) = *d*.

Summarizing, the rule format states that if the agent of the network defined as ⟨*id*, *P*, *V*⟩ enables locally a move, and such a move is permitted by the environmental conditions, then the agent is allowed to evolve and change accordingly the variables under its control.

We point out that, as a special case, ad-hoc actions can be envisioned to model movements to or from communities, which is typical of dynamic scenarios–see, e.g., Aldini, (2022) for a possible semantic characterization. Just notice that such a kind of actions would affect the structure G of the tuple describing the network configuration. As an example, the general semantic rule scheme describing the action of leaving a group is as follows:

$$\left(leave\right)\;\frac{P\overset{leave\text{\_}\mathrm{com}}{\to }P^{\prime }\;\wedge \;cond}{\langle \left\{\langle id,P,V\rangle \right\}\cup S,G,W\rangle \overset{a}{\to }\langle \left\{\langle id,P^{\prime },V^{\prime }\rangle \right\}\cup S,G^{\prime },W^{\prime }\rangle }$$

where *leave*_*com* is the name of such an action, the side condition cond specifies the enabling situation and the identification of the community *G* ∈ *CNames* that the agent *id* is leaving, *V*′ and $W^{\prime }$ express possible updates to the local/global repositories *V* and W due to such a move, and $G^{\prime }=G\left(G\right)\backslash \left\{id\right\}$ represents the update of the involved community. We can reason analogously for a corresponding action *join*_*com* modeling the entry into a community *G*, in which case we have $G^{\prime }=G\left(G\right)\cup \left\{id\right\}$ .

From the cooperation model standpoint, the rule scheme *async* enables forms of knowledge-based communication. Indeed, if the local repository modification (and/or the side condition cond ) depends on some content deriving from the environment, then a data-driven communication from the environment to such an agent is actually modeled. Analogously, writing to the global repository, to which any other agent may have access, represents a form of community-based multicast communication. Sometimes, these forms of (asynchronous) communication are not enough as two (or more) agents have to synchronize over a certain event. To model such a kind of interaction, the following general semantic rule scheme is needed:

$$\left(sync\right)\;\frac{P\overset{a}{\to }P^{\prime }\;Q\overset{b}{\to }Q^{\prime }\;\exists G\in CNames.{id}_{1},{id}_{2}\in G\left(G\right)\;\wedge \;cond}{\langle \left\{\langle {id}_{1},P,V_{1}\rangle ,\langle {id}_{2},Q,V_{2}\rangle \right\}\cup S,G,W\rangle \overset{a\times b}{\to }\langle \left\{\langle {id}_{1},P^{\prime },V_{1}^{\prime }\rangle ,\langle {id}_{2},Q^{\prime },V_{2}^{\prime }\rangle \right\}\cup S,G,W^{\prime }\rangle }$$

where the action *a* × *b* expresses the simultaneous execution of the actions *a* and *b*, so that the two involved agents evolve synchronously. The form of the side conditions is as discussed above, with the additional constraint that the two agents involved in the (synchronous) communication must be members of the same community, which is formally expressed by the predicate $\exists G\in CNames.{id}_{1},{id}_{2}\in G\left(G\right)$ . As a special case, it is possible to define an ad-hoc semantic rule scheme modeling a multicast synchronous communication from an agent of a community to the other agents of the same community–see, e.g., Aldini, (2018) for a possible characterization.

We now discuss the case of a purely synchronous mode of execution, which requires a slightly different approach relying on a two-steps semantics. In the first step, the local actions of the agents that are enabled by the environment according to the given side conditions are determined. In the second step, one action per agent is sampled nondeterministically and the system performs a move by simultaneously executing all the sampled actions. By assuming that the network of agents S includes the agent ⟨*id*, *P*, *V*⟩, the general semantic rule scheme implementing the first step is as follows:

$$\left(global\right)\;\frac{P\overset{a}{\to }P^{\prime }\;\wedge \;cond}{\langle id,P,V\rangle_{\langle S,G,W\rangle }\overset{a}{\to }\langle id,P^{\prime },V^{\prime }\rangle_{\langle \left(S\backslash \left\{\langle id,P,V\rangle \right\}\right)\cup \left\{\langle id,P^{\prime },V^{\prime }\rangle \right\},G,W^{\prime }\rangle }}$$

Notice that in the conclusion of the rule scheme, the triple of elements describing the agent is decorated with the subscripted context expressing the environment with respect to which the side condition must be evaluated. More precisely, the rule scheme *global* expresses whether the network ⟨S,G,W⟩ enables the execution of the action *a* offered in isolation by the agent represented by ⟨*id*, *P*, *V*⟩. This is done through the verification of the side condition cond , parameterized by the elements of the triple ⟨S,G,W⟩ representing the environment of ⟨*id*, *P*, *V*⟩. The rule scheme expresses also what would be the effect of such an execution upon the agent and upon the network. Thus, the same considerations related to the asynchronous case apply as well, the unique difference being that the *global* semantics defines what actions can be potentially performed by the agents in the network. Since every agent is expected to enable at least one action to not block the synchronous evolution of the network, we assume also the following rule:

$$\begin{array}{c} \left(idle\right)\;\frac{\langle id,P,V\rangle_{\langle S,G,W\rangle }\to \;\;\;\;\;\;\;\;\;\;\;\;\;\;}{\langle id,P,V\rangle_{\langle S,G,W\rangle }\overset{\tau }{\to }\langle id,P,V\rangle_{\langle S,G,W\rangle }} \end{array}$$

the effect of which is to allow the agent to stay idle without blocking the network.

Then, in the second step, the network semantics must express the simultaneous execution of one action per agent. By assuming $S={⋃}_{i=1}^{n}\left\langle {id}_{i},P_{i},V_{i}\right\rangle$ , the semantic rule for the second step is as follows:

$$\begin{array}{c} \left(network\right)\;\frac{{⋀}_{i=1}^{n}\langle {id}_{i},P_{i},V_{i}\rangle_{\langle S,G,W\rangle }\overset{a_{i}}{\to }\langle {id}_{i},P_{i}^{\prime },V_{i}^{\prime }\rangle_{\langle S_{i},G,W_{i}\rangle }}{\langle {⋃}_{i=1}^{n}\langle {id}_{i},P_{i},V_{i}\rangle ,G,W\rangle \overset{\tau }{\to }\langle {⋃}_{i=1}^{n}\langle {id}_{i},P_{i}^{\prime },V_{i}^{\prime }\rangle ,G,\prod W_{i}\rangle } \end{array}$$

In practice, in the premise of the rule each agent (indexed by *i*) offers a transition labeled with *a*_*i*_ that derives from the application of the rule scheme *global* or *idle*. Then, the conclusion establishes that all these moves are performed synchronously, as modeled by the *τ* action. ^3^ The proposed scheme is intentionally general. More sophisticated variants of the *network* semantic rule are however possible. For instance, only specific agents (e.g., of selected communities) could be engaged in the synchronization and perform a move. Alternatively, each *a*_*i*_ in the premise may be replaced by a unique action *a*, expressing that the involved agents must synchronize on the specific action. Such a condition may be too strong, as some agents may be not available to execute the action *a*, thus blocking all the others. However, similarly as discussed above, it is sufficient to use an ad-hoc version of the *idle* semantic rule that adds the action information as a negative premise on *a* and decorates the *τ* action with a subscripted *a*. Then, the *network* semantic rule may enable the synchronization of the involved agents that offer either *a* or *τ*_*a*_. These variants emphasize the flexibility and the expressiveness of the approach, which make it adequate to deal with even very specific requirements of various application domains.

In any case, independently from the chosen mode of execution, the semantics of a system ⟨S,G,W⟩ will be given by the smallest LTS with initial state ⟨S,G,W⟩ and transitions deriving from the application of the SOS rules at hand. We observe that the proposed rule schemes express a general format that may potentially guide the definition of a library of several, alternative rules. Such rules can be customized to deal with a comprehensive set of behavioral models and application domains. Obviously, a tradeoff exists between such an expressive power and the efficiency issues that may arise when checking complex side conditions in order to build the underlying LTS.

Example 4.

*Assume a social network* ⟨S,G,W⟩ *of agents adopting the synchronous mode of execution and including the agent* ⟨*id*, *F*, *V*⟩ *of the previous example. One specific instance of the* *global* *rule scheme, which is related to the execution of action* *a* = *nbr* *, may establish that cautious agents (identified by type 2) accept the news whenever the number of neighbours accepting the news is greater than the agent’s threshold. This rule can be formalized easily, first of all by setting the following side conditions:*

$$\left(id.type=2\right)\;\wedge \;\left(id.threshold<\;|\;\;\left\{{id}^{\prime }|{id}^{\prime }\neq id\wedge {id}^{\prime }.accept=true\wedge \exists G.{id}^{\prime },id\in G\left(G\right)\right\}\;\;|\right)$$

*Notice that the neighbours of the agent* *id* *are those agents, different from* *id* *, belonging to communities of which* *id* *is a member. Then, as a side effect, we would also need to update* *V* *with the mapping* *accept* = *true* *, i.e.,* *V*′ = *V*[*accept*↦*true*]*.*

*As another use case, assume that* ⟨S,G,W⟩ *is a trustworthy network including the trustor* ⟨*id*, *T*, *V*⟩ *of the previous example. In such a scenario, let us assume the asynchronous mode of execution. In particular, assume by hypothesis that the action* *snd*_*req* *of the trustor must synchronize with a corresponding action* *rcv*_*req* *of the trustee in the same community of the trustor. Therefore, we need one specific instance of the* *sync* *rule scheme with* *a* = *snd*_*req* *and* *b* = *rcv*_*req* *. Then, if the interaction must be enabled only if the trustor trusts the trustee, we would need a side condition as follows:*

$${id}_{1}.\theta \leq f\left({id}_{1}.\alpha ,{id}_{1}.\beta \right)$$

*where* *f* *is the specific trust function, like, e.g., the probability expectation of the Beta distribution,* $\frac{\alpha }{\alpha +\beta }$ (Jøsang and Ismail, 2002)*. Another instance of such a rule scheme is related to the synchronization involving action* *rec*_*acc* *, the consequence of which would be the update* *α* = *α* + 1 *in the local repository of the trustor. We can argue analogously in the case of action* *rec*_*ref* *and the related update involving* *β* *. Finally, one instance of the rule scheme* *async* *would be associated to the execution of action* *leave*_*com* *. If the agent is expected to leave the community whenever the trustee is not trusted anymore, then a side condition of such a rule would be the predicate* *id* *θ* > *f*(*id*.*α*, *id*. *β*)*. Moreover, two side effects would be given by the corresponding update of the community* G(G) *to which the agent belongs and, possibly, the updates* *α* = *β* = 0*.*

## 3 Model Checking Temporal Properties

The verification of the properties of networks of agents is conducted through model checking (Clarke et al., 1999). Therefore, we need to define a sufficiently expressive and intuitive logic to reason about the various levels of information that our framework can express. To this aim, in this section we present a temporal logic for the specification of properties of networks, which is an instance of action/state-based logics à la CTL (De Nicola and Vaandrager, 1990; ter Beek et al., 2008). The logic is rather standard and its main novelties are concerned with the treatment of the atomic formulas, in a way that recalls and favors the agent/community perspective of the modeling language.

The set of formulas N of the network logic we propose is generated through the following syntax:

$$\begin{array}{c} \Phi \;⩴\;true|id.a|z⋈r|\Phi \wedge \Phi |\neg \Phi |A\pi |E\pi \\ \pi \;⩴\;\Phi \;\;U\;\Phi |\Phi \;U^{\leq k}\Phi \end{array}$$

where:

• r∈R , k∈N , and ⋈ is any arithmetic comparison operator;
• *id*. *a* is the action-based atomic formula, and is satisfied by any state enabling the execution of action *a* ∈ *Act* by agent *id*;
• *z*⋈*r* is the state-based atomic formula, and is satisfied by any state in which the evaluation of variable *z* satisfies the condition ⋈*r*;
• *Aπ* and *Eπ* express the classical universally and existentially quantified path formulas;
• the two flavours of the *until* operator represent the unique type of path formulas; basically a path satisfies Φ_1_ *U* Φ_2_ if it begins with a finite sequence of states satisfying Φ_1_ followed by a state satisfying Φ_2_ (the *k*-bounded version adds a requirement on the length of such a finite sequence).

As mentioned above, the main peculiarities of the logic are given by the atomic formulas, while the composite formulas are standard. The atomic formulas are action-based (*id*.*a*), denoting the execution of an action *a* by the agent *id*, and state-based (*z*⋈*r*), denoting that the state variable *z* satisfies a certain condition parameterized by *r*.

As far as the semantics of the action-based formula *id*. *a* is concerned, we have to distinguish between the two modes of execution. In the asynchronous setting, *id*. *a* holds in ⟨S,G,W⟩ , denoted by ${\left\langle S,G,W\right\rangle ⊧}_{N}id.a$ , if either agent ⟨id,P,V⟩∈S can execute action *a* in ⟨S,G,W⟩ by virtue of a semantic rule of scheme *async*, or agent ⟨id,P,V⟩∈S contributes, by offering action *a*, to the execution of a synchronized action *a* × *b* in ⟨S,G,W⟩ by virtue of a semantic rule of scheme *sync*. In the synchronous setting, *id*. *a* holds in ⟨S,G,W⟩ if agent ⟨id,P,V⟩∈S contributes, by executing action *a* locally, to the execution of the global, synchronous action *τ* enabled in ⟨S,G,W⟩ by virtue of the semantic rule *network*.

As far as the semantics of the state-based formula *z*⋈*r* is concerned, we point out that, in our framework, any state of the LTS modeling a network is labeled with different types of information: the identities of the agents forming the system communities, their local repositories, and the global repositories. Hence, in order to allow for the definition of any kind of state-based requirement, we admit *z* to represent combinations of different types of values filtered in a certain way. To this aim, we distinguish the following three cases.

The first case refers to the state-based formulas over global variables. In this case, let *z*≔*f*{*w* | *ϕ*_*g*_}, such that *f* is a scalar function, *w* ∈ *WNames*, and *ϕ*_*g*_ is a logic formula filtering communities. The intuition is that the values of the global variable *w* taken from those communities that satisfy *ϕ*_*g*_ are combined through *f* to obtain the result *z*. The logic formula *ϕ*_*g*_ obeys the following syntax:

$$\phi_{g}\;⩴\;true|c⋈k|w⋈r|\neg \phi_{g}|\phi_{g}\wedge \phi_{g}$$

where k∈N , *w* ∈ *WNames*, and r∈R . A formula *ϕ*_*g*_ is a Boolean predicate used to select communities based on conditions over their identity (*c*⋈*k*, where *c* stands for community)^4^, conditions over the value of their global variables (*w*⋈*r*), and logical combinations of such atomic conditions. Semantically, the evaluation of *z*≔*f*{*w* | *ϕ*_*g*_} in a network state ⟨S,G,W⟩ is given by:

$$f\left\{\;|\left(W\left(G\right)\right)\left(w\right)\;|\;G\in CNames\;\wedge {\left(W,G\right)⊧}_{g}\phi_{g}|\;\right\}$$ (1)

where *f* works on values of a multiset and the satisfiability relation ⊧_*g*_ for the atomic formulas generated by *ϕ*_*g*_ is defined as follows (the case of the composite formulas is standard):

$$\begin{array}{cc} \left(W,G\right)⊧true & holds\;always\\ \left(W,G\right)⊧c⋈k & \;\mathrm{iff}\;\;G⋈k\\ \left(W,G\right)⊧w⋈r & \;\mathrm{iff}\;\;\left(W\left(G\right)\right)\left(w\right)⋈r \end{array}$$

If the evaluation of *z*≔*f*{*w* | *ϕ*_*g*_} satisfies the condition ⋈*r*, then we have that *z*⋈*r* holds in ⟨S,G,W⟩ , denoted by ${\left\langle S,G,W\right\rangle ⊧}_{N}z⋈r$ . Summarizing, *f* combines the values of the global variable *w* extracted from those communities that satisfy the community predicate *ϕ*_*g*_; then the resulting value is compared to *r*.

The second case refers to the state-based formulas over local variables. In this case, let *z*≔*f*{*v* | *ϕ*_*l*_}, such that *f* is a scalar function, *v* ∈ *VNames*, and *ϕ*_*l*_ is a logic formula filtering agents. The intuition is that the values of the local variable *v* taken from those agents that satisfy *ϕ*_*l*_ are combined through *f* to obtain the result *z*. The logic formula *ϕ*_*l*_ obeys the following syntax:

$$\phi_{l}\;⩴\;true|ide⋈k|ide\in G|v⋈r|v⋈z|\neg \phi_{l}|\phi_{l}\wedge \phi_{l}$$

where k∈N , *G* ∈ *CNames*, *v* ∈ *VNames*, r∈R , and *z*≔*f*{*w* | *ϕ*_*g*_} is any combination of global variables as previously defined. A formula *ϕ*_*l*_ is a Boolean predicate used to select agents based on their identity (*ide*⋈*k*)^5^, community membership (*ide* ∈ *G*), evaluation of their local variables compared to constant values (*v*⋈*r*) or combinations of global variables (*v*⋈*z*), and logical combinations of such atomic conditions. Semantically, the evaluation of *z*≔*f*{*v* | *ϕ*_*l*_} in a network state ⟨S,G,W⟩ is given by:

$$f\left\{{\;|V\left(v\right)\;|\;\left\langle id,P,V\right\rangle \in S\wedge \left\langle id,P,V\right\rangle }_{\left\langle S,G,W\right\rangle }{⊧}_{l}\phi_{l}|\;\right\}$$ (2)

The satisfiability relation ⊧_*l*_ for the atomic formulas generated by *ϕ*_*l*_ is defined as follows (the case of the composite formulas is standard):

$$\begin{array}{cc} \langle id,P,V\rangle_{\langle S,G,W\rangle }⊧true & holds\;always\\ \langle id,P,V\rangle_{\langle S,G,W\rangle }⊧ide⋈k & \;\mathrm{iff}\;\;id⋈k\\ \langle id,P,V\rangle_{\langle S,G,W\rangle }⊧ide\in G & \;\mathrm{iff}\;\;id\in G\left(G\right)\\ \langle id,P,V\rangle_{\langle S,G,W\rangle }⊧v⋈r & \;\mathrm{iff}\;\;V\left(v\right)⋈r\\ \langle id,P,V\rangle_{\langle S,G,W\rangle }⊧v⋈z & \;\mathrm{iff}\;\;V\left(v\right)⋈\left(1\right) \end{array}$$

Notice that, for the semantics of *v*⋈*z*, with *z*≔*f*{*w* | *ϕ*_*g*_}, the evaluation of *v* in ⟨S,G,W⟩ is compared to the evaluation of *z* in the same state, which is computed as stated by Eq. 1. Then, as in the first case, if the evaluation of *z*≔*f*{*v* | *ϕ*_*l*_} in ⟨S,G,W⟩ satisfies the condition ⋈*r*, we have that *z*⋈*r* holds in ⟨S,G,W⟩ . Summarizing, *f* combines the values of the local variable *v* extracted from those agents that satisfy the local predicate *ϕ*_*l*_; then the resulting value is compared to *r*.

The third case is similar to the previous one and refers to the state-based formulas over identities. In this case, let *z*≔*f*{*ide* | *ϕ*_*l*_}. The intuition is that the values of the identities of those agents that satisfy *ϕ*_*l*_ are combined through *f* to obtain the result *z*. Similarly as in the case of Eq. 2, the evaluation of *f*{*ide* | *ϕ*_*l*_} in a network state ⟨S,G,W⟩ is given as follows:

$$f\left\{{id\;|\;\left\langle id,P,V\right\rangle \in S\wedge \left\langle id,P,V\right\rangle }_{\left\langle S,G,W\right\rangle }{⊧}_{l}\phi_{l}\right\}$$ (3)

Notice that *f* applies to identities, which, by their uniqueness, do not form multisets.

Now the semantics for the atomic formulas of N is clarified. Hence, we are ready to define the satisfiability relation, denoted by ${⊧}_{N}$ , for the non-atomic operators of the network logic. For this purpose, given a LTS (*Q*, *q*_0_, *L*, *R*) we need to define the notion of a path. A path *σ* is a (possibly infinite) sequence of transitions of the form:

$$\sigma ≔q_{0}\overset{a_{0}}{\to }q_{1}\ldots q_{j-1}\overset{a_{j-1}}{\to }q_{j}\ldots$$

where $q_{j-1}\overset{a_{j-1}}{\to }q_{j}\in R$ for each *j* > 0. Every state *q*_*j*_ in the path is denoted by *σ*(*j*). Moreover, we denote with *Path*(*q*) the set of paths starting in state *q* ∈ *Q*. The notion of path is needed to formalize the quantified path operators. In particular, the semantics of formula Φ *U* Φ′ states that a path satisfies the formula if it reaches a state that satisfies Φ′, while satisfying Φ in each intermediate state; note that the path could be empty if its initial state satisfies Φ′. As far as the *k*-bounded version of *U* is concerned, an additional condition must be applied, which expresses that the length of the prefix of the path terminating in the state satisfying Φ′ must be ≤k . The formal semantics of the composite operators of our network logic is presented in Table 2.

**TABLE 2:** Satisfiability relation of the network logic.

${q⊧}_{N}\Phi \wedge \Phi^{\prime }$	$iff\;{q⊧}_{N}\Phi \;and\;{q⊧}_{N}\Phi^{\prime }$
${q⊧}_{N}\neg \Phi$	$iff\;q{⊭}_{N}\Phi$
${q⊧}_{N}A\pi$	$iff\;\forall \sigma \in Path\left(q\right):{\sigma ⊧}_{N}\pi$
${q⊧}_{N}E\pi$	$iff\;\exists \sigma \in Path\left(q\right):{\sigma ⊧}_{N}\pi$
${\sigma ⊧}_{N}\Phi \;U\;\Phi^{\prime }$	*iff ∃ i* ≥ 0
	$\sigma {\left(i\right)⊧}_{N}\Phi^{\prime }\wedge \left(for\;all\;0\leq j<i:\sigma {\left(j\right)⊧}_{N}\Phi \right)$
${\sigma ⊧}_{N}\Phi \;U^{\leq k}\;\Phi^{\prime }$	*iff ∃* 0 ≤ *i* ≤ *k*
	$\sigma {\left(i\right)⊧}_{N}\Phi^{\prime }\wedge \left(for\;all\;0\leq j<i:\sigma {\left(j\right)⊧}_{N}\Phi \right)$

Example 5. *Let us consider the social network* ⟨S,G,W⟩ *of the previous example. The following state-based atomic formula* Φ*:*

$$count\left\{ide\;|\;\left(ide\in 1\right)\wedge \left(accept=true\right)\wedge \left(type=2\right)\right\}>3$$

*is true if and only if the number of agents of type 2 belonging to the community 1 of the social network and that are accepting (and sharing) the news, is greater than 3. Then, through formula* *E true U* Φ *we can evaluate whether a state is reachable that satisfies* Φ*.*

*On the other hand, let us consider the case of the trustworthy network example. Given* *n* *the identity of the trustor of interest, the following composite formula* Φ*:*

$$n.leave\text{\_}\mathrm{com}\wedge \left(\min\left\{\alpha \;|\;\left(ide=n\right)\right\}\geq k\right)$$

*checks whether the agent* *n* *is available to leave the community* (*since the action* *n*. *leave*_*com* *is enabled*) *even if the value of its local variable* *α* *is* ≥k *. Again, through formula* *E true U* Φ *we check whether such a state is reachable.*

## 4 Use Cases and Quantitative Extensions

The objective of the proposed framework is to generalize various approaches to the same problem, which differ from each other for the requirements of the application domain. Hence, it would be useful to have a general-purpose approach, with high-level rules and policies, that can be refined and adapted to each specific case.

For example, an instance of the presented general-purpose modeling approach was proposed in previous work (Aldini, 2016, Aldini, 2018), in the specific domain of trustworthy networks, in which trust and reputation models are used to govern the interactions among trustors and trustees. Notice that the examples reported in the previous section illustrate a simplification of a trustor agent and associated behavioral rules. As in such examples, the mode of execution is asynchronous and the most interesting rules are those related to the semantic rule scheme *sync*, as it is used to describe trust-based interactions between agents. More precisely, the side conditions of the semantic rules of such a scheme describe both the trust-based communication policies (e.g., a certain interaction from trustor *A* to trustee *B* is enabled if and only if the trust of *A* towards *B* is higher/lower than the trust threshold applied by *A*) and the policies behind the computation of trust values (e.g., the trust from *A* to *B* is computed by combining several variables, including the dispositional trust of *A*, the previous experience with *B*, and the reputation of *B*). The local repositories include any local trust-based information needed to govern the policies above (e.g., the dispositional trust of *A* towards unknown trustees, the trust threshold applied by *A*, and the scores used to adjust trust after each satisfactory/unsatisfactory interaction). The community-based global repositories are used to collect the opinions shared by the agents within each community to form the reputation scores feeding the trust model.

Then, through model checking, properties expressed in our network logic are used to analyze, e.g., how the trust towards a trustee as perceived by a community is determined depending on the services delivered by such an agent. Variants of such properties allow also to investigate the impact of attacks performed, e.g., by injecting false recommendations. The analysis of real-world case studies, like the Trust-Incentive Service Management by Zhang et al. (2007), the Reputation-based Framework for Sensor Networks by Ganeriwal et al. (2008), and the Robust Reputation System by Buchegger and Boudec, (2004), was conducted automatically through the model checker NuSMV (Cimatti et al., 2002), thanks to a mapping from our specification language to the model of finite state machines used by the software tool.

The proposed modeling approach is general enough to allow for standard extensions to, e.g., probabilistic and stochastic models. For instance, in Aldini, (2022), it is extended with probabilities in order to model and analyze the spread of fake news in social networks. The network is divided into communities of agents, which in turn may exhibit different attitudes to share unchecked news or to conduct some fact checking. The examples reported in the previous section illustrate the non-probabilistic behavior of a type of agent susceptible to stimuli from the environment. The local repositories include the variables characterizing the agent’s attitute to believe, check, and share news.

The reference model underlying the approach of Aldini, (2022) is that of fully probabilistic LTSs (PTSs, for short) obeying the generative model of probabilities (Van Glabbeek et al., 1995). Analogously, our basic calculus is enriched with probabilistic information, similarly as done, e.g., in Baeten et al. (1992). For instance, in *a*. *P* action *a* is executed with probability 1, while the choice operator *P* + *Q* is replaced by the probabilistic choice operator *P* + ^*p*^ *Q*, with $p\in \left.\right]0,1\left[\right.$ , stating that an action of *P* (respectively, *Q*) is chosen with probability *p* (respectively, 1 − *p*). The mode of execution is synchronous: the *global* and *network* semantic rule schemes are extended accordingly to deal properly with such quantitative information in respect of the underlying model of probabilities.^6^.

The verification of PTSs relies on model checking of probabilistic temporal logic formulas (Kwiatkowska et al., 2011; Chen et al., 2013), which are described in a version of our logic that replaces the quantified path operators with the PCTL probabilistic (reachability) operator $P_{⋈p}\left(\pi \right)$ (Hansson and Jonsson, 1994; Bianco and de Alfaro, 1995). The automated analysis was possible through a mapping to the PRISM model checker (Kwiatkowska et al., 2011). The goal of the analysis was to estimate the propagation of fake news over the whole network, depending on the topology of the system and the presence of reliable fact checkers.

In the following, we complete such an overview of potential applications, by considering an example based on another instance of our framework.

### 4.1 Use Case: Blockchain Efficiency

In order to show the flexibility of our approach, here we discuss a case study requiring to deal with stochastically timed events. In such a way, our basic process calculus becomes a stochastic process calculus, in which actions are enriched with rates of exponentially distributed random variables that represent the action duration. Thus, such models give rise to stochastic processes in the form of (action-labeled) Continuous Time Markov chains (Clark et al., 2007). Technically, the operators of our basic calculus are still the same, with the trick of adopting the additional syntax and semantics of the stochastic process algebra PEPA (Hillston, 1996; Tribastone et al., 2009). In particular, actions are pairs of the form (*a*, *λ*), where *a* ∈ *Act* and $\lambda \in R^{+}$ is a positive rate representing the parameter of an exponential probability distribution governing the duration of the timed action. In this setting, the choice operator captures a notion of competition solved via the *race policy*: the action to execute is the one that samples the least duration. We refer to the citations above for all the details about the semantics of stochastic processes. In our use case, we assume the fully asynchronous mode of execution, so that in the following we have to specify the instances of the rule *async* tailored to the given use case.

The objective of the case study is to model a network of peers (P2P network) exchanging information about the blocks of a blockchain, which are generated by special agents called miners - see, e.g., Gamage et al. (2020) for a comprehensive overview of this distributed ledger technology. The blockchain model under consideration is permissionless and based on the proof-of-work mechanism (as in the case, e.g., of Bitcoin). Basically, any peer can mine a new block by solving a cryptographic puzzle called proof-of-work. To this aim, it is essential for the miner to learn information about the most recent block added to the blockchain and the data with which a new block is compiled, which depend on the specific application domain (e.g., virtual currency transactions in the case of Bitcoin). Here, we abstract away from the application domain and we concentrate on the blockchain management.

Peers acting as miners have the following behavioral pattern:

$$\begin{array}{ccc} Miner & \overset{def}{=} & \left(obs\text{\_}\mathrm{block},prop\text{\_}\mathrm{rate}\right).Miner+\left(mine,mining\text{\_}\mathrm{rate}\right).{Miner}^{\prime }\\ {Miner}^{\prime } & \overset{def}{=} & \left(obs\text{\_}\mathrm{block},resume\text{\_}\mathrm{rate}\right).Miner+\left(add\text{\_}\mathrm{block},prop\text{\_}\mathrm{rate}\right).Miner \end{array}$$

A mining node can notice that a new block was mined and propagated through the miner’s community (action *obs_block*) and, at the same time, tries to solve the proof-of-work that would allow him to mine the next block (action *mine*) to be added to the blockchain and propagated to the network (action *add_block*). The other ordinary peers advertise and relay to their reference communities any new block added to the blockchain. Hence, they simply act as forwarder nodes:

$$\begin{array}{ccc} Peer & \overset{def}{=} & \left(obs\text{\_}\mathrm{block},prop\text{\_}\mathrm{rate}\right).Peer+\left(prop\text{\_}\mathrm{block},prop\text{\_}\mathrm{rate}\right).Peer \end{array}$$

A peer can notice that a new block was mined (action *obs_block*) and can propagate newly received blocks (action *prop_block*).

As far as the local repositories are concerned, every node shall maintain a local copy of the blockchain; for the sake of simplicity we limit each node to store the last block of the blockchain, which is abstractedly represented by a local counter *block_id* initially set to 0 for every node of the network. As far as the community-based global repositories are concerned, we use a global variable *last_block_id* storing the most recent block propagated in the community. With such additional information in view–used to define the local mapping *V* of each node and the global mappings W for the communities–we now define the several instances of the semantic rule scheme *async*. For each instance, we specify the action of interest, the enabling conditions, and the side effects:

1. case *a* = *obs*_*block*:
  • ∃G∈CNames:id∈G(G)∧id.block_id<(W(G))(last_block_id)

  • $V^{\prime }=V\left[block\text{\_}\mathrm{id}\mapsto \left(W\left(G\right)\right)\left(last\text{\_}block\text{\_}id\right)\right]$

  • $W^{\prime }=W$

2. case *a* = *prop*_*block*:
  • ∃G∈CNames:id∈G(G)∧id.block_id>(W(G))(last_block_id)

  • *V*′ = *V*
  • $W^{\prime }\left(G\right)=W\left(G\right)\left[last\text{\_}block\text{\_}id\mapsto V\left(block\text{\_}\mathrm{id}\right)\right]$

3. case *a* = *add*_*block*:
  • ∃G∈CNames:id∈G(G)∧(id.block_id+1)>(W(G))(last_block_id)

  • *V*′ = *V*[*block*_*id*↦*V*(*block*_*id*) + 1]
  • $W^{\prime }\left(G\right)=W\left(G\right)\left[last\text{\_}block\text{\_}id\mapsto V\left(block\text{\_}\mathrm{id}\right)+1\right]$

The first case, which refers to the observation of a new block by a node in one of its communities, requires the node to update its local copy of the blockchain. The second case, which refers to the propagation of a new block by a node to one of its communities, requires the node to update the global repository of that community. The third case, which refers to the upload of a new block to the blockchain, requires the miner to update its local copy and to propagate the block. Notice that, by the presence of several potential communities (see the existential quantifier over *G* ∈ *CNames*), such cases may enable several different outgoing transitions, one per involved community. Any other action, like action *mine* in our example, does not require side conditions and/or effects, i.e., the *async* rule scheme is applied with cond≔true and no variation of the local/global repositories.

Essentially, the specification requires just to define the behavioral pattern of the node types (*Miner* and *Peer*) and the pre/post-conditions associated with the execution of the relevant actions. Analogously, we now show through a simple example how it is easy to model properties of interest.

Block propagation delays may potentially impair the correctness of the blockchain sharing process, because a miner could mine and propagate a block before learning of a newly mined block that has been added to the blockchain. Such a misalignment problem is known as blockchain fork. To solve the issue, the network abandons the blocks that are not in the longest chain. Hence, performance and correctness are tightly connected, as the speed at which peers learn of new blocks is related to the likelihood of forks in the blockchain. Recently, in Chandrasekaran et al. (2022) an empirical study of the information propagation delays between nodes in blockchain P2P networks was proposed that emphasizes how the likelihood of forks drastically diminished since 2013. In particular, block propagation delays are estimated in the top four blockchain-based applications, including Bitcoin.

**FIGURE 1:**
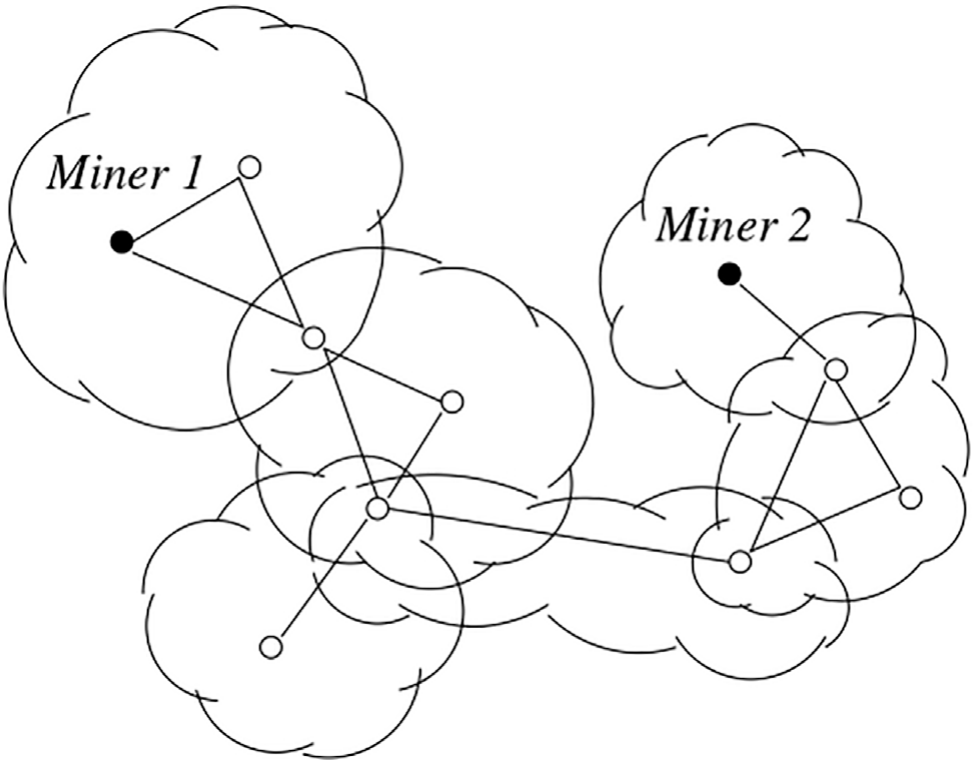
Example of P2P network with 6 communities; some representative peers are depicted, including 2 miners.

Here, we propose a formal and automated verification of the analysis mentioned above, based on the use of the PRISM model checker^7^. For analysis purposes, we decided to instantiate the rates of the timed actions according to the Bitcoin related estimates of Chandrasekaran et al. (2022): the expected time to mine is about 10 min, while the mean (respectively, median) end-to-end propagation delay is about 4 s (respectively, about 0.4 s). Moreover, we modeled various configurations, represented by the topology shown in Figure 1, in which the P2P network radius - represented by the number of involved communities, depicted as clouds - is equal to 6. When a block is advertised in a community, all the members of the community react by experiencing the same delay, so that the overall end-to-end delay of the network depends on the network radius. Two miners are present in the network, while the other peers are either members of a single community or belonging to the intersection of many of them.

For the purpose of model checking, we consider a probabilistic reachability property of the form $P_{⋈p}\left(\pi \right)$ , where *π* is an *until* formula expressing the reachability of a state in which a peer mines a new block and uses it to extend an obsolete version of the blockchain, thus causing a fork. Formally, if we concentrate on the miner with *id* = 1, such a condition is a mixture of action and state based formulas defined as follows:

$$\left(1.add\text{\_}\mathrm{block}\right)\wedge \left(count\left\{ide\;|\;ide=1\wedge block\text{\_}\mathrm{id}<\max\left\{last\text{\_}\mathrm{block}\text{\_}\mathrm{id}\;|\;true\right\}\right\}=1\right).$$

The first conjunct holds when the first miner is enabled to update the blockchain. The second conjunct holds when such an update is obsolete as a more recent block is circulating in the network. We can reason analogously for the other miner, and then join the result of the two properties.

**FIGURE 2:**
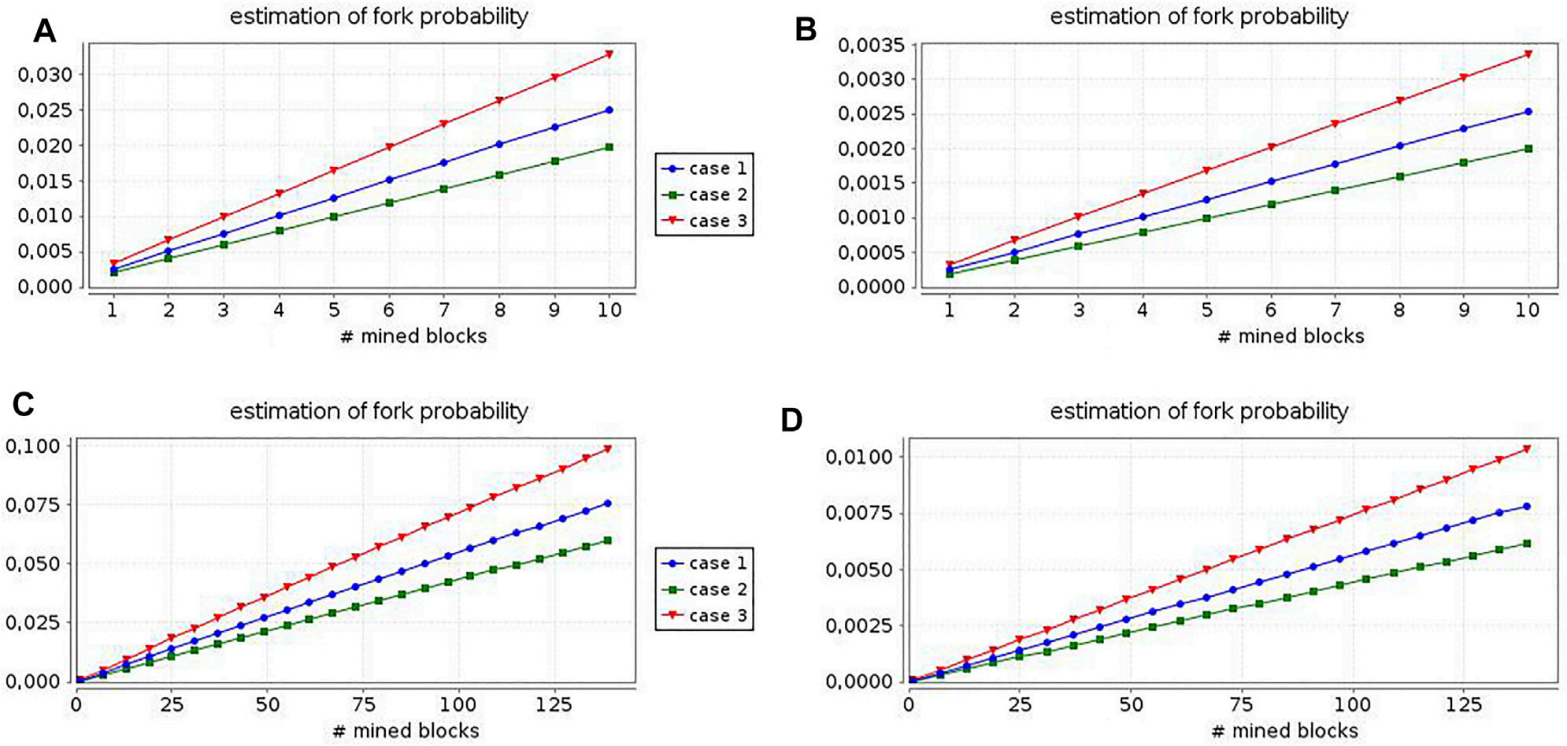
Relation between blockchain length and fork likelihood: analysis for 4 different scenarios.

In Figure 2, we show the results of such an analysis by considering the four combinations deriving from two different configuration choices. The first dimension is given by the topology specification: in scenarios A and B we have exactly the representative nodes depicted in Figure 1, while in scenarios C and D only the miners and the peers in the intersecting areas between the communities are modeled. Then, in the first two scenarios we measure the fork likelihood in a period of time equal to 100 min, while in the other two scenarios we refer to a 1 day interval. The second dimension is given by the propagation delay between each pair of peers, which is chosen to correspond to the mean end-to-end delay measured in Chandrasekaran et al. (2022) for scenarios A and C, and to the median end-to-end delay measured in Chandrasekaran et al. (2022) for scenarios B and D. Moreover, each figure presents the results obtained in three different cases: in case (1) both miners experience the same mining delay (10 min), in case (2) the second miner is slower (15 min), while in the third case the second miner is faster (5 min).

In general, case (1) emphasizes that the fork probability is negligible, especially in cases (b) and (d). These results confirm the performance shown in Chandrasekaran et al. (2022). In detail, cases (2) and (3) reveal that the monitoring of the proof-of-work expected time is critical to maintain the fork likelihood at the desired level. Summarizing, already this simple case study illustrates that our framework is flexible and easy-to-use both from the modeling and the verification standpoints, also in the quantitative setting.

## 5 Related Work and Conclusions

The aim of the proposed approach is to provide a modeling and analysis framework that can be instantiated to specific application domains. The common feature of such domains is that they are characterized by collections of autonomous, dynamic, and interactive agents exhibiting a wide spectrum of cooperation patterns, as well as both reactive and proactive behaviors. The reported examples emphasize that the considered systems may express social relations of human agents in virtual environments, human–computer interactions, and also machine to machine communication. These include online services for smart and sustainable environments, and computer supported cooperative networks.

The verification of coordination and control strategies for cooperative multi-agent systems is of paramount importance even in the setting of inter-robot communications. To this aim, several formal approaches to the design of coordination for robotics emerged in the literature. For instance, the design method proposed in Dai et al. (2016) employs concurrent finite automata and is based on a top-down approach recalling the separation of concerns adopted in our framework at a higher abstraction level. In Gu et al. (2020), the specific problem of synthesising and verifying collision-free paths for autonomous multi-agent systems is dealt with formally through stochastic timed automata and statistical model checking. The verification is conducted automatically through the software tool UPPAAL. In Abd Alrahman and Piterman, (2021), reconfigurable multi-agent systems are modeled via finite automata and model checked using a variant of the Linear Temporal Logic (LTL). The authors emphasize that formal paradigms for modeling dynamic multi-agent systems cannot rely (only) on point-to-point communication. Instead, group-based communication is more appropriate, which is exactly one of the principles behind our framework. By following the same basic ideas, formal modeling paradigms and probabilistic model checking techniques are adopted for the analysis of autonomous agents by Sekizawa et al. (2015) and by Al-Nuaimi et al. (2018). Both approaches use the software tool PRISM for the automated analysis, similarly as done in the quantitative extensions of our framework. In general, all the formal approaches mentioned above rely directly on paradigms that are also at the base of our framework, on top of which we defined a high-level process algebraic specification language. The need for high-level languages in this setting is emphasized, e.g., by De Nicola et al. (2018); Abd Alrahman and Piterman (2021). For instance, we mention the languages ISPL (Lomuscio et al., 2009) and SCEL (De Nicola et al., 2015). The semantics of the former is based on concurrent labeled transition systems, which specifically adopt a form of synchronous communication. Interestingly, model checking is based on an epistemic logic encompassing a knowledge operator. On the other hand, the latter naturally supports knowledge-based communication for dynamic systems, in a way that recalls the method used in our framework to support uni/multi-cast communication via local/global repositories. The full semantics of SCEL is not trivial to export to a runtime environment; tool support is given, e.g., by the model checker SPIN and the MAUDE framework.

In the literature, it is worth mentioning that formal, process-algebraic approaches (Loreti and Hillston, 2016), semi-formal, architectural description approaches (Ozkaya and Kloukinas, 2013), and combinations of both (Basu et al., 2011; Hennicker et al., 2014; Bures et al., 2016) have been proposed to model and analyze dynamic reconfigurable architectures (De Nicola et al., 2020) and (self-)adaptive systems (Gabor et al., 2020). In particular, the language CARMA (Loreti and Hillston, 2016) is specifically defined to model collective adaptive systems and shares several features with our framework, such as the separation of concerns advocated in Section 2, support for local/global views, and a formal semantics in operational style. The process calculus of CARMA is stochastic and has a Markovian semantics, on which numerical analysis based on simulation can be conducted. Moreover, CARMA is equipped with an architectural-style specification language on top of the calculus. By virtue of its modeling capabilities, CARMA is an ideal candidate for representing an instance of the modeling framework proposed in this paper. The BIP framework of Basu et al. (2011) proposes synchronous priority-based communication and a rigorous semantics based on finite-state automata and Petri nets. Compositional verification methods are based on static analysis of local/global invariants. For instance, deadlock-freedom is checked for a robot controller. Interestingly, BIP can be part of a software design flow culminating in deployable code generation. The HELENA approach of Hennicker et al. (2014) formalises the modeling of ensembles (i.e., groups of dynamic collaborating entities) through a class of automata. A mapping towards Promela allows for model checking verification through the SPIN model checker (Klarl, 2015). The modeling of ensembles is also the goal of the DEECo approach of Bures et al. (2016), the operational semantics of which is defined in terms of labeled transition systems. Tool support is provided to enable the verification of reachability properties.

In many of the cases discussed above, classical temporal logics, like LTL and PCTL, support, via model checking, the formal verification of dynamic, multi-agent systems. Sometimes, ad-hoc extensions are used to model specific properties of cyber-physical systems, such as spatial-based conditions (Ciancia et al., 2018; Platzer et al., 2019). The property specification language proposed in our work encompasses the features of CTL-like logics, with a specific emphasis on the separation of concerns and local/global views that characterize the modeling style of our framework.

The key factor of the proposed approach that represents the novelty of this paper is given by the flexibility of a high-level framework combining an action-based formalism with data-driven communication mechanisms based on which different, customized semantics can be provided and supported by several automated tools. So, with respect to the state-of-the-art, by itself the proposed approach does not add new theoretical insights and results. Together with the ease of use in modeling both behavioral patterns and property specifications, the flexibility mentioned above makes our framework adequate to model collective adaptive systems and to support those programming frameworks (Beal et al., 2015; Berndtsson and Mellin, 2018; Casadei et al., 2018) used to develop them.

## Data Availability

The raw data supporting the conclusions of this article will be made available by the authors, without undue reservation.

## Footnotes

1 This can be generalized to consider a separate domain for each variable. By the way, in this paper we assume that *D* is a finite, numerical domain.

2 For the sake of simplicity we a ssume that *WNames* and *VNames* are disjoint.

3 We point out that $\prod W_{i}$ is a shorthand expressing the combination of updates $W_{i}$ applied to the global data repository W by virtue of the moves performed locally by the *n* agents. It is worth noticing that concurrent accesses to the same global variable may occur whenever no mutual exclusion mechanisms are used explicitly by the agents. It is known that this leads to nondeterministic behaviors. This is reflected correctly by the *network* semantic rule, which, in such a case, would enable multiple outgoing transitions, depending on the nondeterminism influencing the way in which W can be updated. However, if the system at hand implements mutual exclusion mechanisms, these would be modeled at the level of the agents’ behavior and of the semantics of the *global* rule scheme, so that no nondeterminism about the update of W would emerge by applying the rule *network*.

4 For the sake of simplicity, here we are assuming that CNames⊆N and the condition *c*⋈*k* applies to the natural *i* representing the community identity, i.e., *i*⋈*k*. If using another domain for community names (e.g., strings) then the elements of the term ⋈*k* would change accordingly.

5 We recall that, similarly as argued for the case of communities identities, we have that agents identities are expressed as naturals. While an obvious condition identifying a specific agent is of the form *ide* = *n*, with n∈N , we could also envision the use of inequality operators if, e.g., the identities are ordered according to some criteria.

6 Models combining nondeterminism and probabilities, like in Markov Decision Processes, can be adopted as well in our approach, by adapting accordingly the semantics.

7 The PRISM source file resulting from our specification is available at: https://github.com/aldinia/prism-bc-specs.
